# Heart Rate and Systolic Blood Pressure Variability on Recently Diagnosed Diabetics

**DOI:** 10.5935/abc.20150073

**Published:** 2015-09

**Authors:** Anaclara Michel-Chávez, Bruno Estañol, José Antonio Gien-López, Adriana Robles-Cabrera, María Elena Huitrado-Duarte, René Moreno-Morales, Brayans Becerra-Luna

**Affiliations:** Instituto Nacional de Ciencias Medicas y Nutrición Salvador Zubirán, México Distrito Federal - Mexico

**Keywords:** Heart Rate, Arterial Pressure, Diabetes Mellitus / diagnosis, Diabetes Complications, Diabetic Neuropathies

## Abstract

**Background:**

Diabetes affects approximately 250 million people in the world. Cardiovascular autonomic neuropathy is a common complication of diabetes that leads to severe postural hypotension, exercise intolerance, and increased incidence of silent myocardial infarction.

**Objective:**

To determine the variability of heart rate (HR) and systolic blood pressure (SBP) in recently diagnosed diabetic patients.

**Methods:**

The study included 30 patients with a diagnosis of type 2 diabetes of less than 2 years and 30 healthy controls. We used a Finapres® device to measure during five minutes beat-to-beat HR and blood pressure in three experimental conditions: supine position, standing position, and rhythmic breathing at 0.1 Hz. The results were analyzed in the time and frequency domains.

**Results:**

In the HR analysis, statistically significant differences were found in the time domain, specifically on short-term values such as standard deviation of NN intervals (SDNN), root mean square of successive differences (RMSSD), and number of pairs of successive NNs that differ by more than 50 ms (pNN50). In the BP analysis, there were no significant differences, but there was a sympathetic dominance in all three conditions. The baroreflex sensitivity (BRS) decreased in patients with early diabetes compared with healthy subjects during the standing maneuver.

**Conclusions:**

There is a decrease in HR variability in patients with early type 2 diabetes. No changes were observed in the BP analysis in the supine position, but there were changes in BRS with the standing maneuver, probably due to sympathetic hyperactivity.

## Introduction

Cardiovascular diseases and type 2 diabetes mellitus (DM) are the main causes of death in the American continent and common causes of disability, premature death, and excessive expenses^1^.

Cardiovascular autonomic neuropathy (CAN) is a common type of autonomic dysfunction in patients with DM and is associated with abnormalities in the control of the heart rate (HR), with loss of its variability, decreased baroreceptor sensitivity (BRS), and late changes in vascular dynamics^2,3^. CAN is detected in about 7% of both types 1 and 2 DM at the time of the diagnosis. The annual increase in prevalence of CAN has been reported to be around 6% in type 2 DM^4-7^.

The prevalence of confirmed CAN (defined as an abnormality in at least two cardiovascular HR results) in clinical studies in unselected populations, including patients with type 1 or 2 DM, varies from 16.6 to 20%^5,8^. This prevalence may increase to 65% with increasing age and DM duration^5,6^. CAN has been linked to tachycardia at rest, orthostatic hypotension, exercise intolerance, increased incidence of asymptomatic ischemia, myocardial infarction, and decreased rate of survival after myocardial infarction^3^.

In healthy individuals, HR has a high inter-beat interval (IBI) variability rate which fluctuates with breathing, increasing during inspiration and decreasing during expiration^9^. The HR variability (HRV) based on IBI variations in short-term or long‑term recordings may be represented, according to the type of mathematical processing, by the HRV analysis in the time domain and frequency domain (spectral analysis)^9,10^.

In short-term recordings, different spectral components may be identified depending on their frequencies in Hz. High-frequency (HF) components are considered an area of vagal influence, whereas low-frequency (LF) components are under sympathetic and some vagal influence, although baroreceptor influences have also been postulated^9^.

Standardized protocols of autonomic load (breathing, ortho-clinostatic test, head-up tilt test) for examination of the HRV spectral analysis in short-term recordings impose a stress element to assess the level and reactivity of both sympathetic and parasympathetic systems^9^.

The objective of this study was to determine beat-to-beat HR and blood pressure (BP) variabilities in patients with type 2 DM with less than two years of diagnosis and compare the results with variabilities in these parameters in healthy subjects.

## Methods

A descriptive, transversal, prolective, comparative, and non-randomized study was developed with individuals of both genders, including 30 diabetic subjects with less than two years from the diagnosis and 30 healthy subjects between 30 and 60 years.

The subjects with DM were identified from a monitoring protocol of a cohort of patients with insulin resistance. During follow-up of these patients, a 75-gram oral glucose tolerance test was performed periodically, and when the results confirmed the diagnosis of DM, the patient was invited to participate in the study. Urinalysis ruled out proteinuria and kidney damage, whereas nerve conduction velocities were performed to rule out somatic peripheral neuropathy. These studies were performed to assess damage to these organs by long-standing hyperglycemia. Finally, the patients were required to have a normal funduscopic exam performed by a certified neurologist to rule out diabetic retinopathy at the time of the study. These variables were determined as control parameters to ensure that the duration of the DM was not too long.

Atherosclerotic peripheral vascular disease was absent in all patients, based on a carotid intima-media thickness below 0.685 mm (0.659-0.691 mm). This is the range established by the CARMELA study (2011) for the maximum age of the patients included in our study (60 years)^11^.

The project was approved by the Ethics and Research Committee of the institution and was in compliance with the Declaration of Helsinki. Before testing, each participant signed an informed consent form.

Prior to the study, the cases had capillary blood glucose levels between 60 mg/dL and 200 mg/dL. We requested the subjects in both groups to be free from any stimulant substances 24 hours prior to the study and to have at least 8 hours of sleep the night before. We excluded patients with underlying diseases with an autonomic component or of any other nature which could interfere with the test results.

Measurement of the two variables of interest – BP and IBI – was carried out with Finometer® (Finapres®, the Netherlands), during three maneuvers (registered for five minutes each one):

1. Supine position.
2. Standing after one minute of stabilization.
3. Rhythmic breathing of 6 cycles per minute paced electronically.

The data obtained from the time series of IBI in milliseconds (ms) and systolic BP (SBP) in mmHg (for each heart beat) in these three conditions were analyzed with time diagram, histogram, autoregressive analysis, and fast Fourier transform.

The time series were manually cleaned from artifacts or premature beats. A statistical analysis was performed in the time and frequency domains using the SPSS software. Mean, standard deviation (SD), standard error and coefficient of variation for the time domain were obtained. Using Beatscope (Finometer’s own program to extract the time series of IBI in ms and BP in mmHg), the time series were transferred to Excel for statistical evaluation. For the spectral analysis, we used SPSS, MatLab, Kubios, and Nevrokard. The level of statistical significance was set at p ≤ 0.05.

## Results

There were no significant differences between the two groups in the variables age, gender, SBP, and fasting glucose (Table 1). Body mass index (BMI) was slightly higher in the diabetic group compared with the control group.

**Table 1:** Demographic variables

Variable	Diabetic n = 30	Healthy n = 30	p
Age	40.5(38-48)	39(35-42)	0.065
Female	14(46%)	14(46%)	1
BMI (Kg/m2)	27.01(25.8-29.38)	25.95(24.5-27.36)	0.006
SBP (mmHg)	117(109-120)	110(110-120)	0.94
Fasting glucose (mg/dL)	99.33(82-105)	79.12(72-83)	0.66
Carotid intima-media thickness (mm)			
Right	0.56(0.53-0.66)	-	-
Left	0.57(0.54-0.66)	-	-
Months from diagnosis	10.8	-	-
Diagnostic method for DM2 (OGTT)	30(100%)		

BMI: Body mass index; SBP: Systolic blood pressure; DM2: Type 2 diabetes mellitus, OGTT: Oral glucose tolerance test.

None of the patients presented abnormal sensory or motor peripheral nerve conduction velocities, F responses or H reflexes. Sympathetic skin responses were present with normal amplitudes and latencies.

**Figure 1:**
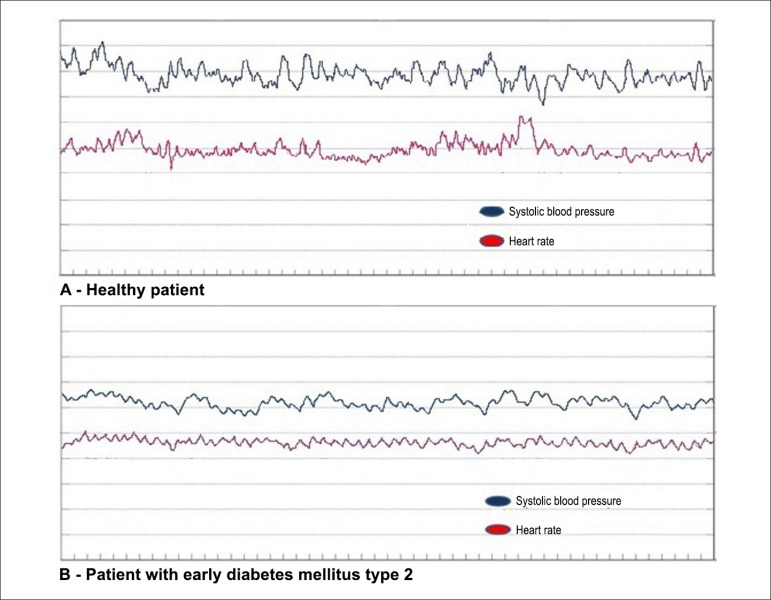
Histogram in the supine position

In the first maneuver, the patient was placed in the supine position for five minutes after one minute of stabilization. In healthy subjects, HR and BP had relatively low variability, without sudden changes in frequencies (Figure 1A). This was due to the fact that the variability observed in this position was solely attributed to breathing. Patients with early DM (Figure 1B) showed a slightly lower HR variability compared with the control subjects. The BP related to body changes while in the supine position was also slightly decreased.

In the second maneuver, the subjects were asked to stand up after a 5-minute supine rest. Immediately after standing up, the BP decreased and the HR increased as a consequence of baroreceptor action. These variables returned to their baseline levels within approximately 30 seconds, reaching their highest point at 15 seconds. This has been documented as the 15/30 index or Ewing score, a normal physiological phenomenon.

Rhythmic breathing started with the aid of a visual metronome with 5-second inspirations and 5-second exhalations during a recording time of 5 minutes. This condition resulted in graphs with more regularity and wider variations in BP and HR, showing an integrity of the baroreflex in both cases. However, there was again a lower variability in diabetic compared with healthy subjects.

When we conducted the numerical analysis, we obtained punctual values that allowed the comparison of the variables established for our objectives.

The HR values in the supine position are summarized in Table 2.

**Table 2:** Heart rate in the supine position

	Diabetes	Control	p
HR (mean)	67.3 ± 1.69	66.71 ± 1.77	0.82
HR (SD)	2.5 ± 0.15	3.61 ± 0.26	< 0.01
SDNN (ms)	28.9(25.6–37.1)	43.6(31.6–53.9)	< 0.01
RMSSD (ms)	24.55(20–30.7)	24.3(24.3–50.5)	0.03
pNN50 (%)	3.55(0.7–11.2)	9.5(1.3–33.5)	0.04
TP (ms2)	717(530–1289)	1324.5(803–2854)	< 0.01
LF (ms2)	175.5(110–305)	302(327–620)	0.01
HF (ms2)	215(70–278)	299(186–712)	0.01
LF (nu)	58.8 ± 17.66	48.96 ± 18.35	0.31
HF (nu)	46.2 ± 17.66	51.03 ± 18.41	0.31

HR: Heart rate; SD: Standard deviation; SDNN: Standard deviation of NN intervals; RMSSD: Root mean square of successive differences; NN50: Number of pairs of successive NNs that differ by more than 50ms; pNN50: Proportion of NN50 divided by the total number of NNs; TP: Total power; LF: Low frequency; HF: High frequency; nu: Normalized units.

Although no differences in the mean HR in beats per minute were found between the two groups, there were significant differences in the SD of these means, the SD of the NN intervals (SDNN), the root mean square of successive differences (RMSSD), and the NN50 percentage (pNN50). In all cases, the values were higher in healthy subjects compared with diabetics , reflecting a higher variability in the control group. When the total power (TP) and its components LF and HF were analyzed, values were significantly higher in healthy subjects, with a predominance of LFs. After normalizing these data, we found no difference between the groups.

Table 3 summarizes the data obtained for HR in the standing position.

**Table 3:** Heart rate in the standing position

	Diabetes	Control	p
HR (mean)	75.78 ± 11.41	78.5 ± 10.93	0.35
HR (SD)	3.14 ± 0.93	5.22 ± 1.91	< 0.01
SDNN (ms)	31.25(23.6–41.1)	47.05(32.6–62)	< 0.01
RMSSD (ms)	20.5(13.7–24.1)	26.05(21.8–34.5)	< 0.01
pNN50 (%)	1.55(0–3.6)	5.25(1.9–14.6)	< 0.01
TP (ms2)	785(409–1270)	2030.5(938–3026)	< 0.01
LF (ms2)	199(122–388)	551.5(357–1130)	< 0.01
HF (ms2)	124(64–210)	322.5(229–542)	< 0.01
LF (nu)	63.27 ± 18.64	61.19 ± 20.59	0.69
HF (nu)	36.77 ± 18.64	38.8 ± 20.59	0.69

HR: Heart rate; SD: Standard deviation; SDNN: Standard deviation of NN intervals; RMSSD: Root mean square of successive differences; NN50: Number of pairs of successive NNs that differ by more than 50 ms; pNN50: Proportion of NN50 divided by the total number of NNs; TP: Total power; LF: Low frequency; HF: High frequency; nu: Normalized units.

The stimulus from the position modification generated changes in the HR, with differences observed only in SD, SDNN, RMSSD, and p-NN50 with higher values for control subjects. In TP, we observed that the proportions cited in the previous Table were maintained. However, on a global level, slightly higher values were observed, with a prevalence of LF both in absolute values as in normalized units (nu).

Table 4 shows the results obtained for HR during rhythmic breathing of 6 cycles per minute. This condition repeated the same findings observed in the supine and standing positions, confirming decreases in SD, SDNN, RMSSD, and pNN50 values as a result of a lower short-term variability in diabetics.

**Table 4:** Heart rate during rhythmic breathing

	Diabetes	Control	p
HR (mean)	70.51 ± 7.43	70.22 ± 10.31	0.91
HR (SD)	4.78 ± 1.82	7.44 ± 7.44	< 0.01
SDNN (ms)	54.7(44.4–72.3)	86.65(68–86.65)	< 0.01
RMSSD (ms)	28.45(19.8–42.1)	51.5(41.2–77.9)	< 0.01
pNN50 (%)	8.4(1.2–20.2)	30(16–46.5)	< 0.01
TP (ms2)	2966.5(1510–4815)	7406(3612–12,098)	< 0.01
LF (ms2)	2136.5(963–3663)	6454(2363–9527)	< 0.01
HF (ms2)	243(65–431)	640(320–1389)	< 0.01
LF (nu)	90.7(84.4-94.2)	88.05(84–91.4)	0.379
HF (nu)	9.3(5.8-15.6)	11.95(8.6–16)	0.340

HR: Heart rate; SD: Standard deviation; SDNN: Standard deviation of NN intervals; RMSSD: Root mean square of successive differences; NN50: Number of pairs of successive NNs that differ by more than 50 ms; pNN50: Proportion of NN50 divided by the total number of NNs; TP: Total power; LF: Low frequency; HF: High frequency; nu: Normalized units.

In the spectral analysis, a significantly higher TP was found in healthy compared with diabetic subjects. It should be noted that the LFs were higher both in punctual values in ms^2^, as well as in *nu*, when compared with the values obtained in the standing and supine positions. The spectral analysis regarding predominance of LF or HF suggests either a sympathetic or parasympathetic predominance.

In the supine position, we found no differences in the SBP parameters (Table 5).

**Table 5:** Systolic blood pressure in the supine position

	Diabetes	Control	p
SBP (mean)	116.27 ± 22.01	107.79 ± 10.37	0.06
SBP (SD)	4.43(3.50-5.95)	4.99(3.46-5.80)	0.73
SBP (max)	130.83 ± 24.35	124.2 ± 15.48392	0.22
SBP (min)	105.2 ± 23	96.26667 ± 13.34924	0.09
TP (mmHg2)	4791.3(3192-7207.5)	5920.8(3074.9-8881.7)	0.44
LF (mmHg2)	1360.8(1095.5-2724.7)	1971.65(1006.1-3508.3)	0.45
HF (mmHg2)	216.31(118.06-536.68)	310.069(204.90-428.18)	0.38

SBP: Systolic blood pressure; SD: Standard deviation; TP: Total power; LF: Low frequency; HF: High frequency.

When the standing maneuver was performed (Table 6), SBP readings showed a higher mean SBP and a higher maximum/minimum pressure ratio in diabetic patients when compared with healthy subjects. No differences in the SD of the SBP were observed between groups. In spite of these findings, TP was higher in controls than in diabetic subjects, with a predominance of LF.

**Table 6:** Systolic blood pressure in the standing position

	Diabetes	Control	p
SBP (mean)	123.19 ± 30.09	107.84 ± 12.99	0.01
SBP (SD)	5.33(4.25–6.53)	5.63(4.42–7.14)	0.75
SBP (max)	140.2 ± 32.79	123.86 ± 4.79	0.01
SBP (min)	107.73 ± 29.45	92.33 ± 15.14	0.01
TP (mmHg2)	2966.5(1510–4815)	7406(3612–12,098)	< 0.01
LF (mmHg2)	2136.5	6454(2363–9527)	< 0.01
HF (mmHg2)	243(65–431)	640(320–1389)	0.20

SBP: Systolic blood pressure; SD: Standard deviation; TP: Total power; LF: Low frequency; HF: High frequency.

Results of the analysis of SBP with rhythmic breathing are summarized in Table 7. Persistent predominance of higher pressures in diabetic patients is be noticed, along with maximum and minimum SBP values. However, in contrast to the previous Table, no statistical significance was found for TP and its components LF and HF.

**Table 7:** Systolic blood pressure with rhythmic breathing

	Diabetes	Control	p
SBP (mean)	128.55 ± 27.98	113.83 ± 15.43	< 0.01
SBP (SD)	7.4(5.8–8.2)	6.7(5.82–7.53)	0.30
SBP (max)	147.16 ± 29.2	132 ± 16.87	< 0.01
SBP (min)	108.8 ± 28.09	97 ± 16.26	0.04
TP (mmHg2)	11,244(8027.5)	10,770(7890–14,281)	0.66
LF (mmHg2)	7450.6(4607.2–12,095)	6843.75(4662.3–9817.4)	0.41
HF (mmHg2)	723.06(301.28–907.92)	632.27(414.05–883.69)	0.77

SBP: Systolic blood pressure; SD: Standard deviation; TP: Total power; LF: Low frequency; HF: High frequency.

The BRS expresses the sensitivity in the response of the IBI to increasing or decreasing SBP. A significant difference was observed in BRS in the standing position, which was lower in the diabetic group compared with that obtained in the control group.

## Discussion

During an initial subclinical stage, CAN is detected through abnormalities in the domains of frequency and time of the HRV spectral analysis and in BRS tests. These abnormalities can even be present at the time of the diagnosis of DM^12^. As CAN progresses, parasympathetic denervation is followed by compensatory sympathetic overdrive, resulting in abnormal cardiac autonomic reflex tests followed by symptomatic CAN. At a stage in which sympathetic denervation of the blood vessels is occurring, autonomic dysfunction correlates clinically with postural hypotension^12^. The time scale for the progression is unclear, but it is estimated that many patients with subclinical CAN will develop features of cardiac involvement within 5 years of developing abnormal parameters in the frequency and time domains^13^.

Ziegler et al.^14^ showed in a meta-analysis an increased mortality among diabetic patients with autonomic neuropathy compared with those without this neuropathy. The risk rate for silent myocardial ischemia in the group with autonomic neuropathy was 1.96 (1.53–2.51; p<0.001). When they analyzed the mortality rate in 2900 subjects, the relative risk of death in patients with DM and autonomic neuropathy was 2.14 (1.83–2.51; p<0.0001)^14^. These data have great importance and suggest that autonomic testing should be an integral part of the approach in all diabetics.

Although these studies have included diabetic patients, most have been conducted in patients with at least 5 years of disease or with chronic diseases, such as peripheral neuropathy, nephropathy, and retinopathy^15^. It has been suggested that the earliest indicator of diabetic autonomic neuropathy is CAN^16^.

Although HRV has been commonly assessed as part of the evaluation of cardiac autonomic neuropathy, the variability in BP has been much less studied^17^. There are only a few studies conducted to analyze specifically the *short-term* (beat-to-beat variability) HRV values in the time domain, or including spectral analysis to assess the sympathetic or parasympathetic influence in the frequency domain.

Exploratory studies at different stages of the disease are important for the understanding of its natural course. In this study, age, gender, basal BP, and fasting glucose were statistically similar in both groups, although there was a slight difference in BMI, which was higher in the diabetic group. This is explained by a higher prevalence of overweight in type 2 diabetic patients (Table 1).

### Heart rate analysis

With the patient in a supine position, there is no stimulus to determine a dominance of the sympathetic over the parasympathetic system. There is no gravity influence and activation of baroreceptors is only determined by changes in BP mediated by respiratory movements. In this first maneuver, a statistically significant difference was found in the SD of the HR of diabetics compared with control subjects. This indicates that the variability in patients with DM is lower than that registered in healthy subjects. This is confirmed in the short-term IBI (in ms) analysis with calculation of the SD of this record (SDNN), root mean square of successive differences (RMSDD), number of times that this consecutive interval from one heart beat to another exceeds 50 milliseconds (NN50), and percentage of these events with regard to the complete series of registered heart beats (pNN50). There is also a greater TP in this maneuver, dominated by LF over HF. By normalizing these units, the bias produced by very low frequencies (VLF) is eliminated. No practical value has been defined for VLF^18^. No statistical significance was also found between *nu* values.

In the standing position, a physical challenge is imposed by gravity which produces a momentary sudden fall in BP that never exceeds 40 mmHg and a compensatory tachycardia lasting approximately 15 seconds. This maneuver activates sympathetic pathways, so when there is damage to either autonomic branch, an imbalance is detected in the described measurements. It has been established in diabetic patients that parasympathetic fibers to the heart are the first to suffer some damage, so it is possible to find early tachycardia in diabetic patients compared with healthy subjects^19^.

We found no differences in mean frequencies between diabetics and healthy subjects. However, in all other short-term variables such as SDNN, RMSSD, and pNN50, we found lower values in the diabetic compared with the control group, indicating a lower HRV. In this maneuver, and for both cases, there was a predominance of a sympathetic over a parasympathetic effect, which may be explained by the fact that in early CAN stages there is still no important sympathetic damage to cause sympathovagal imbalance. This probably indicates that short-term analysis factors (SDNN, RMSSD, and pNN50) might be very useful for initial screening of CAN in these patients, even if the regulatory or sympathovagal balance is intact. This suggests that the loss of variability in patients with DM may be the earliest manifestation of CAN.

During rhythmic breathing (at 0.1Hz), there was a predominance of LF (0.1–0.15Hz), implying a sympathetic challenge. The differences in the parameters were the same as those observed with the previous maneuver. However, here we noticed an important dominance of LF over HF, which indicates that the stimulus of rhythmic breathing conferred this sympathetic property. There was no difference between groups in *nu*.

Hence, we found a lower HRV in patients with early DM when compared with healthy subjects, mainly due to parasympathetic damage to the heart, although early sympathetic damage also occurs, but to a lesser extent (Tables 2-4).

### Systolic blood pressure analysis

BP is mainly determined by cardiac output times peripheral resistance. The sympathetic innervation of the heart controls the HR, ventricular contractility, and stroke volume. Although the cardiovagal innervation does not influence contractility and stroke volume, it has a powerful influence on the HR either by inducing bradycardia or transient tachycardia with the withdrawal of its activity. Blood vessels have a vasomotion at approximately 0.1Hz (each 10 seconds) mediated solely by the sympathetic innervation. The HF variability of the BP is probably transmitted only by the heart, whereas the LF variability is related to the LF variability of the heart and the LF variability due to vasomotion induced by the sympathetic nervous system. Therefore, in the spectral analysis of the BP, there is a sympathetic or LF predominance. This influence is seen in patients with atrial fibrillation in whom HRV (either HF or LF) is absent, but a variability in LF is maintained by the BP^20^.

Baseline pressure in our experimental and control groups was within the normal range and did not differ significantly between each other.

In the supine position, we obtained values with the Finapres® for mean SBP, SD, maximum and minimum pressures, TP, and LF and HF that were not significantly different. It is important to highlight that there were no differences between groups in the baseline analyses, which makes them comparable in regards to the challenges imposed by the sympathetic stimulation (standing position and rhythmic breathing). It should be mentioned that the BP was slightly higher in the diabetic group than in the control group, due to a dominance of the sympathetic control (LF).

During the standing maneuver, SBP was higher in diabetic patients, both in mean, as well as in maximum and minimum numbers. However, the dominance still respected the sympathetic factor of LFs, and this response was even greater in the control group. This might indicate that the vascular response is preserved in the control group, which has better mechanisms of BP regulation, whereas in diabetic patients, sympathetic hyperactivity to the vasculature is not completely balanced by the baroreceptor reflex.

In the rhythmic breathing maneuver, we observed a sustained oscillation of inspiration and expiration in which a sympathetic stimulus dominated over a parasympathetic one, not excluding the control of the latter by mechanisms of BRS. In this maneuver, the only statistical significance was found in the mean SBP in diabetics compared with controls, derived predominantly from the fact that the DM group had higher maximum pressures than the control group. In the frequency domain, TPs and its components LF and HF did not differ significantly between groups. This occurs when there is maintained sympathetic and parasympathetic integrity and is considered a normal response.

In the supine position, the only difference was the lower LF values in diabetics compared with controls, whereas LF/HF rates were similar. This lower response is due to the absence of a gravitational stimulus.

This pattern remained in the standing position, showing no difference between pressure and variability by SD. A significant difference was obtained in TP established by a dominance of LFs in which the control group showed higher values because of better responses. However, this normal response to sympathetic dominance stimulation persists and does not affect the values measured in mmHg.

In rhythmic breathing, the stimulus of the sympathetic effect dominated over the parasympathetic one, but in a similar proportion as in the two previous maneuvers; once again, this did not modify the mean BP, nor its SD. The above shows that the sympathovagal control is intact in both groups, with broader responses in the frequency domain in the control compared with the diabetic group.

### Baroreflex sensitivity analysis

As previously described, the analysis of the BRS derives from a quotient between the IBI (expressed in ms) and the SBP (in mmHg). This reflects a cardiac response to changes in registered pressure and involves both sympathetic control for positive chronotropic effects in face of a BP decrease, as well as parasympathetic control for negative chronotropic effects in face of a BP increase and requirement for IBI prolongation. It is, therefore, important to point out the response to these two major maneuvers, supine position and standing position.

Our results demonstrate that there was no difference in baseline measurements in the supine position, but there was a difference in the standing maneuver, in which control individuals showed a greater sensitivity when compared with diabetic patients. This is because the SBPs were higher in diabetics, and since this variable is calculated as a quotient, it tends to a smaller number. These findings could suggest a slightly lower parasympathetic response in diabetics compared with controls.

## Conclusions

There is a decreased HRV in the supine and standing positions and during rhythmic breathing in diabetics subjects with less than 2 years of diagnosis compared with control subjects, determined by the SD and by the analysis of short-term variabilities [HR (SD), SDNN, pNN50 (%)], probably associated with a parasympathetic failure.

With *nu*, HR spectral values were similar in both groups. In standard values (no *nu)*, the results were significant. It is possible that this loss of variability occurs with a relatively normal sympathetic balance, which suggests that short-term variables may be useful for the assessment of the variability in conditions of good autonomic balance. Therefore, it is suggested that these variables may be the first to become abnormal in the DM spectrum.

There was no significant difference in SBP in diabetic patients with recent diagnosis in the analysis of the time and frequency domains. However, SBP in the standing position tended to be higher in diabetics compared with controls, despite not reaching pathological values.

During orthostatism, BRS was slightly decreased in diabetic subjects with recent diagnosis compared with controls. This contrasts with the results in the supine position, in which there was no difference, probably due to the absence of gravitational stress.

### Limitations of the study

The analysis shows that the HRV is decreased in patients with recent-onset DM. This finding is relevant because only a few studies on HRV have been performed in patients with recent-onset DM, showing that cardiac autonomic neuropathy appears early in the disease. The control of BP is more complex because it involves vagal and sympathetic activity to the heart and sympathetic activity to resistant vessels. The sympathetic damage to blood vessels is probably preserved in the early stages of the disease and may be affected only in late stages. Therefore, more studies or different techniques are necessary to demonstrate changes in BP with analysis of either the time or frequency domains in recent-onset DM. There seems to be sympathetic hyperactivity to the resistant blood vessels in early DM, as shown by the increase in BP in the standing position, although more studies are required to elucidate this point.
